# Putative virulence factors of *Corynebacterium pseudotuberculosis* FRC41: vaccine potential and protein expression

**DOI:** 10.1186/s12934-016-0479-6

**Published:** 2016-05-16

**Authors:** Karina T. O. Santana-Jorge, Túlio M. Santos, Natayme R. Tartaglia, Edgar L. Aguiar, Renata F. S. Souza, Ricardo B. Mariutti, Raphael J. Eberle, Raghuvir K. Arni, Ricardo W. Portela, Roberto Meyer, Vasco Azevedo

**Affiliations:** Departamento de Biologia Geral, Instituto de Ciências Biológicas, Universidade Federal de Minas Gerais, Avenida Antonio Carlos, 6627, Pampulha, Belo Horizonte, 31270-901 Brazil; Uniclon Biotecnologia, Belo Horizonte, MG Brazil; Multiuser Center for Biomolecular Innovation, Instituto de Biociências, Letras e Ciências Exatas, Universidade Estadual Paulista “Júlio de Mesquita Filho”, São José Do Rio Preto, SP Brazil; Laboratório de Imunologia e Biologia Molecular, Instituto de Ciências da Saúde, Universidade Federal da Bahia, Salvador, BA Brazil

**Keywords:** *Corynebacterium pseudotuberculosis*, Pathogenicity and virulence, Vaccine potential, Epitope prediction, Protein expression, Protein purification

## Abstract

**Background:**

*Corynebacterium pseudotuberculosis*, a facultative intracellular bacterial pathogen, is the etiological agent of caseous lymphadenitis (CLA), an infectious disease that affects sheep and goats and it is responsible for significant economic losses. The disease is characterized mainly by bacteria-induced caseous necrosis in lymphatic glands. New vaccines are needed for reliable control and management of CLA. Thus, the putative virulence factors SpaC, SodC, NanH, and PknG from *C. pseudotuberculosis* FRC41 may represent new target proteins for vaccine development and pathogenicity studies.

**Results:**

SpaC, PknG and NanH presented better vaccine potential than SodC after in silico analyses. A total of 136 B and T cell epitopes were predicted from the four putative virulence factors. A cluster analysis was performed to evaluate the redundancy degree among the sequences of the predicted epitopes; 57 clusters were formed, most of them (34) were single clusters. Two clusters from PknG and one from SpaC grouped epitopes for B and T-cell (MHC I and II). These epitopes can thus potentially stimulate a complete immune response (humoral and cellular) against *C. pseudotuberculosis*. Several other clusters, including two from NanH, grouped B-cell epitopes with either MHC I or II epitopes. The four target proteins were expressed in *Escherichia coli*. A purification protocol was developed for PknG expression.

**Conclusions:**

In silico analyses show that the putative virulence factors SpaC, PknG and NanH present good potential for CLA vaccine development. Target proteins were successfully expressed in *E. coli*. A protocol for PknG purification is described.

**Electronic supplementary material:**

The online version of this article (doi:10.1186/s12934-016-0479-6) contains supplementary material, which is available to authorized users.

## Background

Caseous lymphadenitis (CLA) is a chronic, pyogenic, contagious disease of sheep and goat that imposes considerable economic losses for farmers in many countries [1, 2]. The disease is caused by *Corynebacterium pseudotuberculosis* (*C. pseudotuberculosis*): a gram-positive pleomorphic, non-capsulated, non-motile, fimbriated, facultative intracellular bacterium, multiplying within macrophages [1]. *Corynebacterium ulcerans and C. pseudotubercu1osis* produce phospholipase D (PLD), which is unique among corynebacteria. It promotes the hydrolysis of ester bonds in sphingomyelin in mammalian cell membranes, possibly contributing to the spread of the bacteria from the initial site of infection to the secondary sites within the host. Moreover, it provokes dermonecrotic lesions; and at higher doses it is lethal to a number of different species of laboratory and domestic animals [3–5].

CLA disease is expressed in external and visceral forms, either separately or together [3–5]. External CLA lesions appear initially as abscesses that convert later on to pyogranulomas ranging in size from millimeters to centimeters. These external lesions are mostly located within superficial lymph nodes, but infrequently in subcutaneous tissues. Wool or hair over CLA lesions may be lost due to the weak dermonecrotic action of *C. pseudotuberculosis* exotoxins and the pressure atrophy of overlying skin by the lesions. Visceral lesions are not detectable clinically but express themselves according to their number, site and effect on the involved organ. Progressive weight loss, respiratory disorders and chronic recurrent ruminal tympany are the most prominent signs that may accompany visceral CLA lesions.

Identification/removal of infected animals is a key factor for success of disease control measures. Vaccination of healthy animals is another strategy broadly recommended for disease control. In fact, control of CLA depends on vaccination in most countries [2, 5–7]. Although bacterin, toxoid, combined, and live vaccines are available, the disease has persisted even after prolonged vaccination, indicating the suppressive nature of CLA vaccination [5, 7]. *C. pseudotuberculosis* infection of farmer animals can contaminate meat and milk, putting consumers at risk due to its zoonotic potential [7]. The ability of *C. pseudotuberculosis* to infect both animals and humans makes necessary the development of new vaccines for a reliable control and management of CLA once the currently available commercial vaccines are unable to fully protect susceptible animals against the disease [7, 8]. In this way, the study of other *C. pseudotuberculosis* virulence factors that might be involved in CLA pathogenesis can provide new vaccine targets.

The complete genome sequence of a *C. pseudotuberculois* strain (FRC41) isolated from a 12-year-old girl with necrotizing lymphadenitis allowed the identification of *spaC* and *nanH* as genes encoding proteins regarded as potential virulence factors [8]. SpaC is a putative adhesive pili tip protein. The pilus structure can probably make the initial contact with host cell receptors to enable additional ligand-receptor interactions and to facilitate the efficient delivery of virulence factors and intracellular invasion [9]. NanH, by its turn, is a putative extracellular neuraminidase [8]. Neuraminidases, or sialidases, belong to a class of glycosyl hydrolases that catalyze the removal of terminal sialic acid residues from a variety of glycoconjugates and can contribute to the recognition of sialic acids exposed on host cell surfaces. Most sialidase-producing microorganisms are pathogenic or commensal when in close contact with mammalian hosts. It has been also suggested that, in some types of pathogenic bacteria, sialidases function as potential virulence factors that contribute to the recognition of sialic acids exposed on the surface of the host cell [10]. A homologous counterpart of *C. pseudotuberculois* FRC41 NanH was characterized in *C. diphtheriae* KCTC3075 and shown to be a protein containing neuraminidase and trans-sialidase activities [11].

The *C. pseudotuberculosis* FRC41 genome also encodes a putative secreted copper,zinc-dependent superoxide dismutase (SodC) that is characterized by a lipobox motif and may be anchored in the cell membrane [8]. The extracellular location of this enzyme suggests that it may protect the surface of *C. pseudotuberculosis* cells against superoxide generated externally by the mammalian host cells. In *Mycobacterium tuberculosis*, SodC contributes to the resistance of this microorganism against the oxidative burst products generated by activated macrophages [12, 13]. The protective activity of Cu,Zn-SODs has been associated with virulence in other bacteria, such as *Neisseria meningitides* and *Hemophylus ducreyi* [8].

As part of important cell signaling mechanisms, eukaryotic-like serine/threonine protein kinases encountered in bacteria are a class of molecules that also deserves attention since they are part of complex signaling pathways and play a diversity of physiological roles in developmental processes, secondary metabolism, cell division, cell wall synthesis, essential processes, central metabolism, and virulence [14, 15]. *Mycobacterium tuberculosis* genome encodes 11 eukaryotic-like serine/threonine protein kinases (PknA to PknL, except for PknC). Protein kinase G (PknG) gained particular interest because it affects the intracellular traffic of *M. tuberculosis* in macrophages. Most microbes and nonpathogenic mycobacteria quickly find themselves in lysosomes, where they are killed. By contrast, *M. tuberculosis* stays within phagosomes; the bacterium releases PknG to block phagosome-lysosome fusion. Bacteria lacking *pknG* gene are rapidly transferred to lysosomes and eliminated [16, 17]. The genome of *C. pseudotuberculosis* FRC41 has a gene encoding for a putative PknG protein [8] but its function in the bacterium still needs to be investigated.

Therefore, *C. pseudotuberculosis* SpaC, NanH, SodC, and PknG proteins may play important roles in virulence and pathogenicity. In the present work, a characterization and evaluation of the vaccine potential of these proteins were performed in silico. The heterologous expression of these putative virulence factors in *Escherichia coli* is also described.

## Methods

### Protein sequences

The amino acid sequences of the target proteins were retrieved from NCBI GenBank: SpaC [gb| ADK29663.1], SodC [gb| ADK28404.1], NanH [gb| ADK28179.1], PknG [gb| ADK29622.1].

### Homology searches

NCBI BLASTP [18] searches in UniProtKB database [19] were performed to identify homologues of the target proteins in the CMNR group of microorganisms (from *Corynebacterium*, *Mycobacterium*, *Nocardi,* and *Rhodococcus* genera): *Corynebacteriumn, taxid:1716; Mycobacterium, taxid:1763; Nocardia, taxid:1817; Rhodococcus, taxid:1827*. Likewise, BLASTP searches in UniprotKB database were performed to identify homologues of the target proteins in mammalian species of the *Ovis* (taxid: 9935), *Bos* (taxid: 9903), *Equus* (taxid: 9789), *Equus* (taxid: 35510), Mus (taxid: 10088), *Mus* (taxid: 862507) genera and in *Homo sapiens* (taxid: 9606). BLAST Genome [18] searches in *C. pseudotuberculosis* (taxid: 1719) complete genomes available at NCBI genome database were performed to identify the presence of the target protein genes in other *C. pseudotuberculosis* strains.

### Primary and secondary structure analysis, subcellular localization and prediction of protective antigens

ProtParam [20] and Self-OPtimized prediction method with alignment—SOPMA [21] of expasy server were used to analyze different physiological and physicochemical properties of the target proteins. Molecular weight, theoretical pI, amino acid composition, extinction coefficient, estimated half-life, instability index, aliphatic index and grand average of hydropathicity (GRAVY) were calculated using the ProtParam preset parameters. Solvent accessibility, transmembrane helices, globular regions, bend region, random coil and coiled-coil regions were predicted using SOPMA default parameters. The amino acid sequences were evaluated by PSORTb 3.0.2 [22] to predict subcellular localization of the target proteins. SignalP 4.1 [23] was used to predict the presence and location of signal peptide cleavage sites in the amino acid sequences. The method incorporates a prediction of cleavage sites and a signal peptide/non-signal peptide prediction based on a combination of several artificial neural networks. VaxiJen 2.0 [24] was used for alignment-independent prediction of protective antigens. The tool was developed to allow antigen classification solely based on the physicochemical properties of proteins without the need of sequence alignment.

### B-cell epitope prediction

Linear B-cell epitopes were predicted from the target protein sequences using physicochemical properties [25] estimated by in silico methods available in DNASTAR Protean program (Madison, Wisconsin). The Jameson–Wolf method [26] was used to predict the potential antigenic determinants by combining existing methods for protein structural predictions. The results appear as multiple peaks in the antigenic index plot, with each peak signifying a potential antigenic determinant. The emini surface probability method [27] was used to predict the probability that a given region lies on the surface of a protein. The Kyte–Doolittle hydropathy method [28] predicts regional hydropathy of proteins from their amino acid sequences. Hydropathy values are assigned for all amino acids and are then averaged over a user defined window. The average is plotted at the midpoint of the window. The charge density method predicts regions of positive and negative charge by summing charge over a specific range of residues. DNASTAR developed this method using the pK tables of White et al. [29]. Since charged residues tend to lie on the surfaces of proteins, this method aids in predicting surface characteristics. Several wet lab experiments revealed that the antigenic portions were situated in beta turn regions of a protein [30] for these regions the Chou and Fasman beta turn prediction method was used [31, 32]. The Karplus–Schulz flexibility method [33] predicts backbone chain flexibility. The method is useful for resolving antigenic sites, as these regions tend to be among the most flexible in a polypeptide sequence. Conserved domains in the target proteins were identified by searching NCBI’s conserved domain database (CDD) [34]. The results of each method were presented in a graphical frame. The peak of the amino acid residue segment above the threshold value (we used the default) is considered as predicted B-cell epitope. User can select any physicochemical property or a combination of two or more properties for epitope prediction. [35]. We selected amino acid segments in the target protein sequences where peaks above threshold overlapped in four or more methods. B-cell epitopes located in signal peptide or conserved domains were discarded.

### T-cell epitope prediction

MHC I binding prediction was performed using the immune epitope database (IEDB) MHC I binding tool [36] and consensus [37] as prediction method which combines predictions from ANN aka NetMHC (3.4), SMM and comblib methods. Mouse MHC alleles (H-2-Db, H-2-Dd, H-2-Kb, H-2-Kd, H-2-Kk, H-2-Ld) and a peptide length of nine mer were selected to make the predictions from target proteins sequences. A median percentile rank of the four predictions methods was the Consensus representative percentile rank used to select the top 1 % of peptides. A small numbered percentile rank indicates high affinity.

MHC II binding predictions for target proteins were performed using NetMHCII 2.2 server [38] to predict binding of 15 mer peptides to two mouse MHC II alleles (H-2-IAb and H-2-IAd) using artificial neuron networks. The prediction values were given in nM IC50 values, and as a  %-Rank to a set of 1,000,000 random natural peptides. Strong and weak binding (SB, WB) peptides were indicated in the output. T-cell epitopes located in signal peptide or conserved domains were discarded.

### Epitope clustering

Epitope clustering was performed using the IEDB Epitope cluster analysis tool [36]. Clustal omega [39] was used to group predicted B and T-cell epitopes into clusters of similarity based on multiple sequence alignment and visual inspection. Clustal omega alignments were used to double check if single-sequence clusters generated by IEDB epitope cluster analysis tool were in fact composed of unique epitopes (no pairs).

### Cloning procedures

Miniprep plasmid purifications, agarose gel electrophoresis, and *E. coli* media were as described [40]. Amino acids 2–23 and amino acids 2–31 were removed from *sodC* and *nanH* ORF sequences, respectively. These regions containing signal peptide were eliminated before cloning in order to improve protein expression since they are relatively rich in hydrophobic amino acids. ORF codons of all four target proteins were replaced by *E. coli* preferential codons [41]. Optimized ORF sequences were synthesized and individually cloned into pD444-NH expression vector (T5 promoter, IPTG inducible, strong ribosome binding site, His-tag, ampicillin resistance marker, high copy origin of replication, 4027 bp size) by DNA2.0 (Menlo Park, CA). Each ORF-containing plasmid (pD444-NH;*pknG*, pD444-NH;*spaC*, pD444-NH;*sodC*, and pD444-NH;*nanH*) was transformed into BL21(DE3) *E. coli* strains according to the OverExpress™ Electrocompetent Cells kit (Lucigen, Middleton) instructions.

### Protein expression in *E. coli*

Protein expression protocol was according to OverExpress™ Electrocompetent Cells kit (Lucigen, Middleton) instructions. Briefly, transformed cell cultures at OD 0.5–0.7 were induced with 1 mM IPTG for 5 h at 37 °C. SDS-PAGE of non-induced and induced cell culture samples and Coomassie blue staining was as described [42].

### Purification of PknG

Bacteria transformed with pD444-NH;*pknG* was induced as described above. Cell pellet was collected by 8000 rpm centrifugation, resuspended in buffer A (10 mM NaH_2_PO_4_ pH7.4, 300 mM NaCl, 1 % glycerol, 5 mM imidazole), lysed on ice with ten 15-s sonication pulses using a ultrasonic processor Marconi-MA 103 (Piracicaba, São Paulo) and centrifuged at 15,000×*g* for 15 min. The supernatant containing recombinant proteins was purified under native conditions using 1 mL of immobilized Ni Sepharose (GE Healthcare). The resin was washed using buffer A with 80 mM imidazole. Recombinant PknG was eluted from the column with buffer A containing 400 mM imidazole. The eluted protein was dialyzed against buffer B (10 mM NaH_2_PO_4_ buffer pH 7.4 and 50 mM NaCl) and concentrated by ultrafiltration. The concentrated fraction was injected on a Superdex 75 10/300 GL (GE Healthcare) size exclusion column previously equilibrated with buffer B. The purity of the sample was assessed by SDS–PAGE.

## Results and discussion

Traditional vaccination approaches are based on complete pathogen either live attenuated or inactivated. Among the major problems these vaccines brought are crucial safety concerns, because those pathogens being used for immunization may become activated and cause infection. Moreover due to genetic variation of pathogen strains around the world, vaccines are likely to lose their efficacy in different regions or for a specific population. Novel vaccine approaches like DNA vaccines and epitope based vaccines have the potential to overcome these barriers to create more effective, specific, strong, safe and long lasting immune response without all undesired effects [43]. Next-generation sequencing and proteomic techniques have enabled researchers to mine entire microbial genomes, transcriptomes and proteomes to identify novel candidate immunogens [44]. In silico techniques are the best alternative to find out which regions of a protein out of thousands possible candidates are most likely to evoke immune response [35]. This reverse vaccinology approach has enjoyed considerable success in the past decade, beginning with *Neisseria meningitides*, and continuing with *Streptococcus pneumonia*, pathogenic *E. coli*, and antibiotic resistant *Staphylococcus aureus* [44].

### Homology searches

The conservation level between target proteins and proteins of the CMNR group of microorganisms was evaluated by NCBI BLASTP [18] searches in UniprotKB database [19]. This kind of analysis is important for the development of vaccines once they can be used not only for *C. pseudotuberculosis* FRC41 but for other pathogen strains and pathogens of other species. NCBI BLAST Genome searches show the presence of the target protein genes in all 37 *C. pseudotuberculosis* strains currently available in NCBI complete genomes database (data not shown). This indicates that SpaC, SodC, NanH and PknG can potentially be expressed not only in a few strains demonstrating the importance of these proteins for this pathogenic bacterium. Well conserved homologous of the target proteins were also found in microorganisms of the CMNR group (Additional files 1, 2 and 3). These findings are a good indication that a vaccine against *C. pseudotuberculosis* made from the putative virulence factors can be effective not only against numerous strains of the pathogen but also against bacterial pathogens from other species.

The conservation degree among target proteins and mammalian (*Ovis*, *Bos*, *Equus* and *Mus* genera, *Homo sapiens*) proteins was also evaluated by BLASTP searches. The analysis was important to reveal the conservation degree among pathogen proteins and host proteins and so the possibility of undesirable immunological cross-reactions which may induce autoimmunity. The results (Additional files 1, 2 and 3) show that *C. pseudotuberculosis* FRC41 SpaC, SodC, NanH, and PknG sequences share low identity (30 % in average) with mammalian sequences. BLASTP alignments show that most of this weak homology is in conserved domains (data not shown). Thus, regions away from signal peptides and conserved domains are ideal targets for vaccine development.

### Primary and secondary structure analysis

The next step was to evaluate the primary and secondary structure features of SpaC, SodC, NanH and PknG as they can predict stability and reveal functional characteristics of the proteins at some extent. Based on ProtParam instability index, SodC was considered the least stable while PknG was the most stable (Table 1). PknG was also the most hydrophilic with the highest GRAVY (−0.211). This same protein also presented the highest aliphatic (92.91) index (Table 1). SOPMA program, used to calculate secondary structure features of the target proteins, reported that SpaC, SodC and NanH were dominated by random coils, consisting in 45.35, 41.26 and 39.05 %, respectively (Table 2). Alpha helix prevailed (44.06 %) in PknG. The differences in secondary structure content and aliphatic character helps to explain the stability indexes estimated for the target proteins. [45].

**Table 1:** Physicochemical properties of the target proteins estimated using ProtParam

Physicochemical property	SpaC	SodC	NanH	PknG
Number of amino acids	796	206	694	749
Molecular weight	85,964.9	21,099.3	74,683.3	83,349.4
Theoretical pI	5.13	5.96	5.05	5.13
Total number of negatively charged residues (Asp + Glu)	96	23	102	101
Total number of positively charged residues (Arg + Lys)	77	17	79	76
Extinction coefficient^a^	93,085	4595^b^	77,600	81,375
Abs 0.1 % (=1 g/l), assuming all pairs of Cys residues form cystines	1.083	0.218^b^	1.039	0.976
Extinction coefficient^a^	92,710	4470^b^	77,350	81,250
Abs 0.1 % (=1 g/l), assuming all Cys residues are reduced	1.078	0.212^b^	1.036	0.975
*The estimated half-life*				
Mammalian reticulocytes, in vitro	30 h	30 h	30 h	30 h
Yeast, in vivo	>20 h	>20 h	>20 h	>20 h
*Escherichia coli*, in vivo	>10 h	>10 h	>10 h	>10 h
Instability index (II)	28.21 (stable)	19.62 (stable)	32.92 (stable)	38.18 (stable)
Aliphatic index	80.16	71.65	72.58	92.91
Grand average of hydropathicity (GRAVY)	−0.442	−0.245	−0.485	−0.211

^a^ Extinction coefficients are in units of M^−1^ cm^−1^, at 280 nm measured in water

^b^ This protein does not contain any Trp residues. Experience shows that this could result in more than 10 % error in the computed extinction coefficient

**Table 2:** Secondary structure content in the target proteins estimated using SOPMA

Secondary structure	SpaC (%)	SodC (%)	NanH (%)	PknG (%)
Alpha helix (Hh)	111 is 13.94	56 is 27.18	228 is 32.85	330 is 44.06
3_10_ helix (Gg)	0.00	0.00	0.00	0.00
Pi helix(Ii)	0.00	0.00	0.00	0.00
Beta bridge (Bb)	0.00	0.00	0.00	0.00
Extended strand (Ee)	244 is 30.65	39 is 18.93	128 is 18.44	114 is 15.22
Beta turn (Tt)	80 is 10.05	26 is 12.62	67 is 9.65	56 is 7.48
Bend region (Ss)	0.00	0.00	0.00	0.00
Random coil (Cc)	361 is 45.35	85 is 41.26	271 is 39.05	249 is 33.24
Ambiguous states (?)	0.00	0.00	0.00	0.00
Other states	0.00	0.00	0.00	0.00

### Subcellular localization and prediction of protective antigens

The candidate molecules from a eukaryotic pathogen expected to induce immunity comprise proteins that are as follows: (i) present on the surface of the pathogen, (ii) excreted/secreted from the pathogen and (iii) homologous to known proteins involved in pathogenesis and virulence [46]. Signal peptide presence and subcellular localization (Table 3) of SpaC (cell wall), SodC (cytoplasmic membrane) and NanH (extracellular) was as predicted before [8]. They were predicted as protective antigens by VaxiJen. Membrane and secreted proteins are considered potential vaccine targets once they are at the host-pathogen interface. These proteins may interact more directly with host molecules for cell adhesion, invasion, multiplication, immune response evasion, damage generation to the host, and survive to host cell defenses [8, 47, 48].

**Table 3:** Subcellular localization, signal peptide, and prediction of protective antigen for the target proteins

Parameter (program)	SpaC	SodC	NanH	PknG
Subcellular localization (Psortb)	Cell wall (matched LPXTG; score 9.97)	Cytoplasmic Membrane (matched 61246116: superoxide dismutase Cu–Zn precursor; score 9.68)	Extracellular (matched 585539: sialidase precursor EC 3.2.1.18 NEURAMINIDASE; score 9.70)	Cytoplasmic, (matched 54041713: probable serine/threonine-protein kinase pknG; score 9.89)
Signal peptide (signalp 4.1)^a^	No (D = 0.162 D-cutoff = 0.420)	Yes position: 1–35 (cleavage site between pos. 35 and 36: DSA-DK D = 0.631 D-cutoff = 0.450 networks = signalp-TM)	Yes position: 1–31 (cleavage site between pos. 31 and 32: APA-TL D = 0.562 D-cutoff = 0.450 networks = signalp-TM)	No (D = 0.106 D-cutoff = 0.420)
Prediction of protective antigens (VaxiJen)	Probable ANTIGEN (score 0.6912)	Probable ANTIGEN (score 0.7663)	Probable ANTIGEN (score 0.6967)	Probable NON-ANTIGEN score 0.3686)

^a^ For signal peptide prediction, D-cutoff values were set as sensitive (reproduce SignalP 3.0’s sensitivity)

Like its counterpart in *M. tuberculosis*, which is predominantly found soluble in the cytoplasm [15], PknG was predicted as a cytoplasmic protein (Table 3). However, VaxiJen predicted this *C. pseudotuberculosis* putative serine/threonine protein kinase as non-antigenic. In fact, cytoplasmic proteins have not been widely considered as potential immunogens, since they do not have a close contact to many immune systems’ intermediates [49]. Regardless of this, it has been demonstrated that cytoplasmic proteins can be effectively exposed to MHC presentation and may have a key role in the development of a suitable protective immunity. In order to overcome the problem of endogenous antigen access to the MHC II compartment, lysosomal-associated membrane proteins (LAMPs), major lysosomal membrane glycoproteins that contain a cytoplasmic tail targeting sequence that directs the trafficking of the molecule through an endosome/lysossome pathway, including cellular compartments where it is co-localized with MHC II molecules, have been used to induce antigen-trafficking to MHC II compartments and increase the immune response to those antigens [50]. This strategy has shown to elicit enhanced long-term memory response against HIV-1 Gag protein. Besides, a novel mechanism of specific CD8^+^ T cell-mediated protective immunity can recognize malaria proteins expressed in the cytoplasm of parasites, form clusters around infected hepatocytes, and protect against parasites [51]. This strongly indicates that cellular and molecular mechanisms underlying the protective immune responses against intracellular parasites need further studies.

### Linear B-cell epitope prediction

The general problem in achieving an effective treatment of *C. pseudotuberculosis* infections in animals and humans is probably related to the facultative intracellular lifestyle of this bacterium, as it can survive and multiply in macrophages [52]. The knowledge on the immunity induced by *C. pseudotuberculosis* indicates that the resistance to infection is a complex process involving components of the non-specific and specific host responses, in which humoral and cellular immune responses are both operative [7].

B-cell epitopes can induce both primary and secondary immunity. Although it is believed that the majority of B-cell epitopes are conformational epitopes, experimental determination of epitopes has focused primarily on the identification of linear (non conformational) B-cell epitopes [25]. This is mainly because predictions of conformational epitopes depend on experimentally determined protein structures or homologous protein structures for in silico modeling. So far, there is no protein structure of the target proteins or structures of highly homologous proteins available for modeling.

**Fig. 1:**
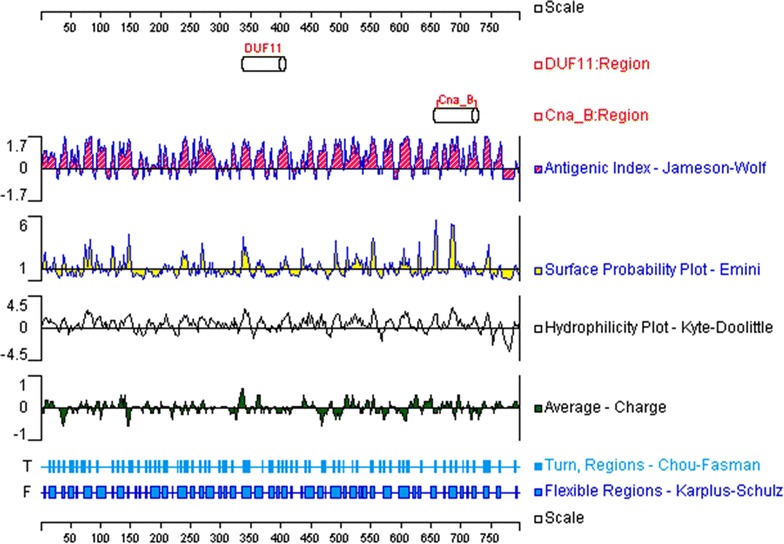
Graphical outputs of the different methods used to quantitate the physicochemical properties used to predict B-cell epitopes from SpaC. On *top* are the conserved domains of the target protein identified by searching NCBI’s Conserved Domain Database (CDD). The *scales* indicate the amino acid positions

**Fig. 2:**
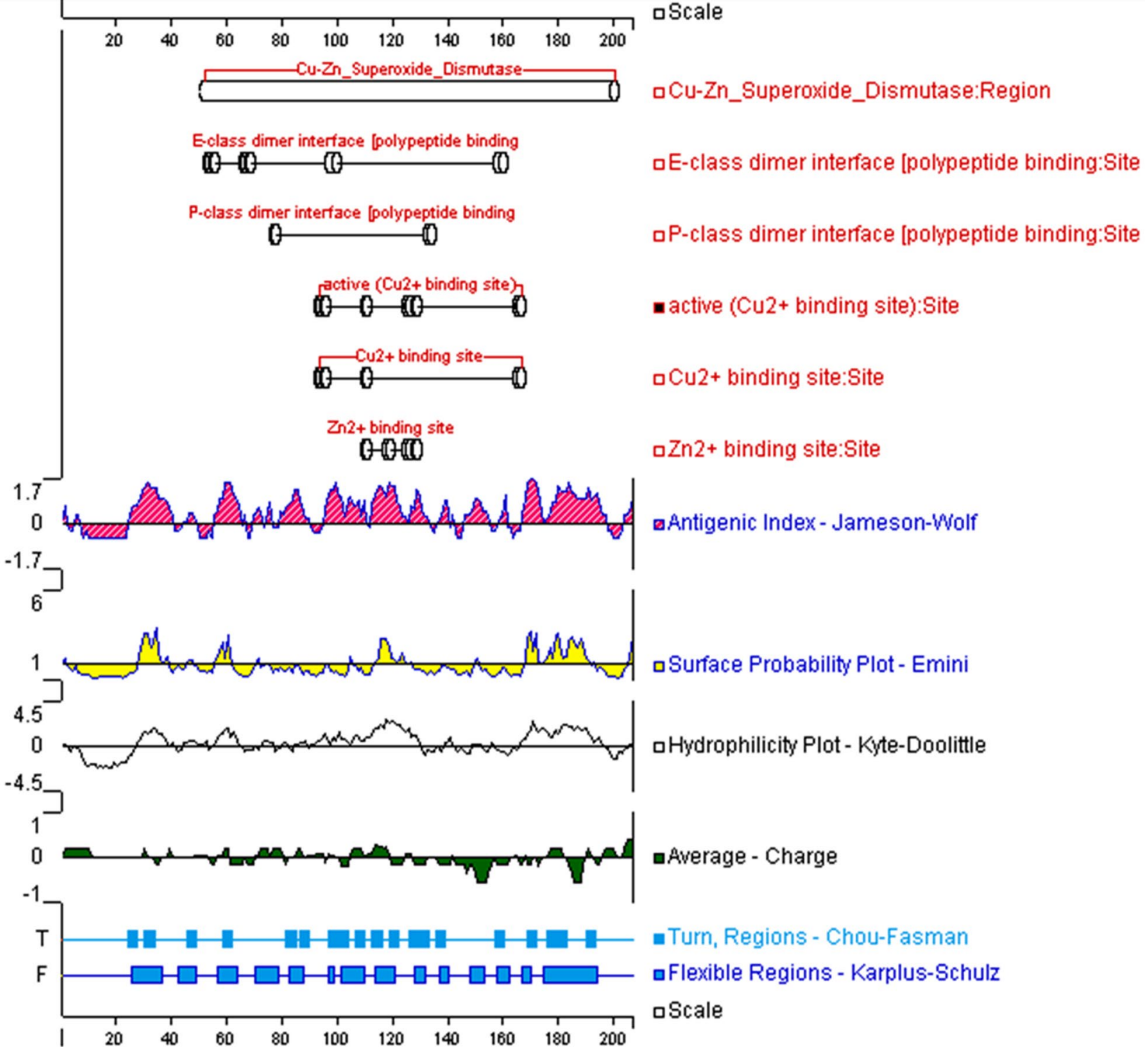
Graphical outputs of the different methods used to quantitate the physicochemical properties used to predict B-cell epitopes from SodC. On *top* are the conserved domains of the target protein identified by searching NCBI’s Conserved Domain Database (CDD). The *scales* indicate the amino acid positions

**Fig. 3:**
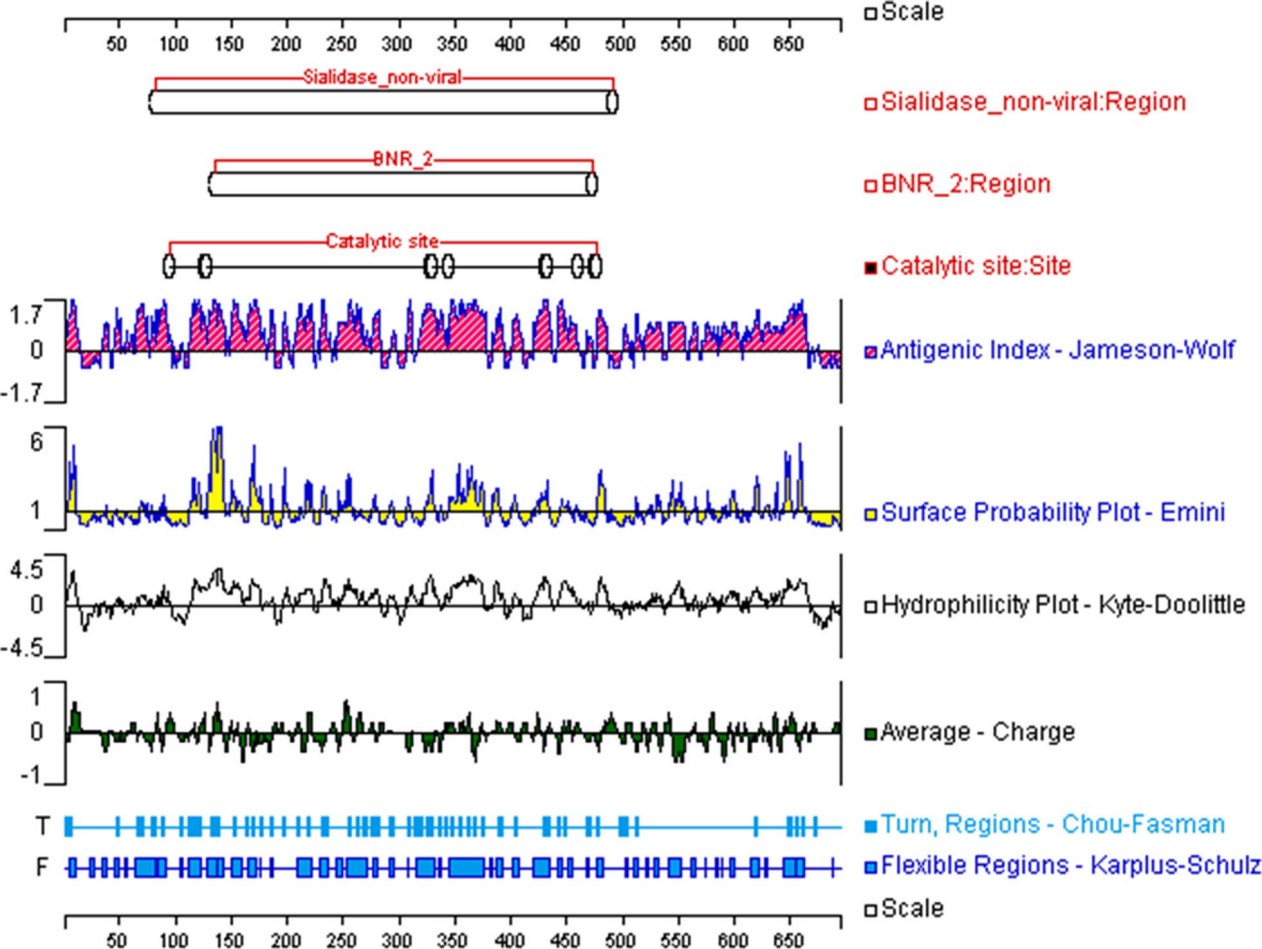
Graphical outputs of the different methods used to quantitate the physicochemical properties used to predict B-cell epitopes from NanH. On *top* are the conserved domains of the target protein identified by searching NCBI’s Conserved Domain Database (CDD). The *scales* indicate the amino acid positions

**Fig. 4:**
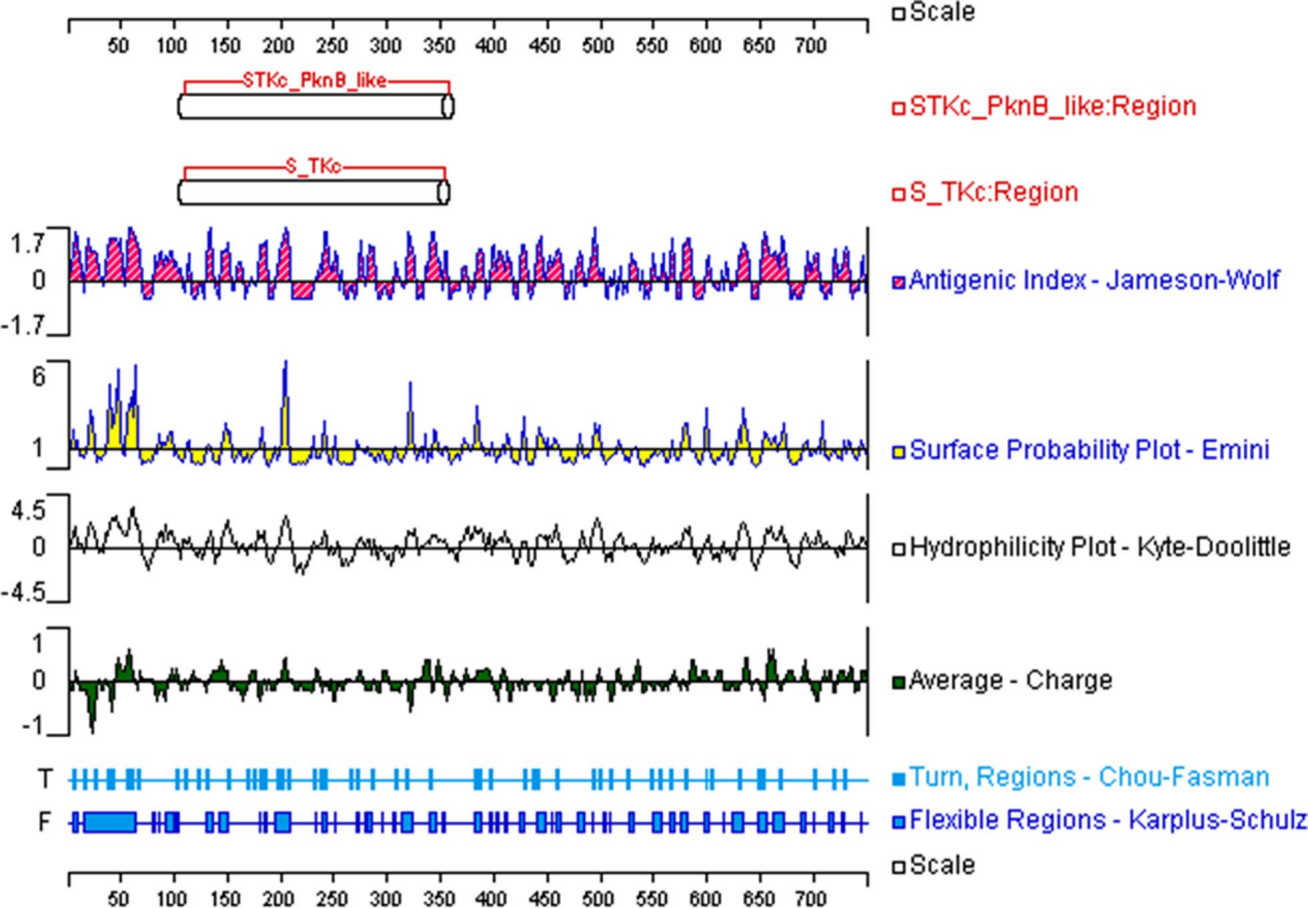
Graphical outputs of the different methods used to quantitate the physicochemical properties used to predict B-cell epitopes from PknG. On *top* are the conserved domains of the target protein identified by searching NCBI’s Conserved Domain Database (CDD). The *scales* indicate the amino acid positions

Most of the existing linear B-cell epitope prediction methods are based on physicochemical properties relating to surface exposure, such as flexibility or hidrophilicity [25, 35], as it is thought that epitopes must lie at the protein surface for antibody binding to occur. Thus, the target proteins were scanned for B-cell epitopes using several methods designed to quantitate protein physicochemical properties. Graphical outputs of the prediction methods are shown in Figs. 1, 2, 3, and 4. High values gave rise to peaks, whereas valleys correspond to negative properties of the protein. Selected B-cell linear epitopes of target proteins are shown in Table 4. The putative adhesive pili tip protein SpaC, seconded by PknG, presented the highest number of B-cell epitopes. We did pick only one B-cell epitope from SodC since the protein is short (206 aa), has a 35 aa long signal peptide (Table 3) and its highly conserved domain occupies most of the amino acid sequence (Fig. 2).

**Table 4:** B-cell epitopes predicted from target proteins

Target Protein	Epitope number	B-cell epitopes^a^
SpaC	1	1-MEVPEKTKVEIRFQTGSKISTPSTPSV-27
SpaC	2	70-SQHTNRGETFNDRNSTDLYVQ-90
SpaC	3	116-AYNPKEGYIYAISQGRLKTLQSSKLRIYDEDPNYPAGHLL-155
SpaC	4	234-NDYTSTGKTDSNYVWGI-250
SpaC	5	251-KNSSNPAVLERIDVRDGSRKEFSLDGVKDPLGQNVEKGIYGT-292
SpaC	6	331-IVAKRKGPTSQNNDATSNG-349
SpaC	7	434-KATYKVTANQSISNNEKCLQNTASIYAN-461
SpaC	8	504-GNGLRKVTYKIEVKNPKGFPETKYSLTDTPQFADSV-539
SpaC	9	540-KLERLKVISDYGKKNQEVQAADISV-564
SpaC	10	615-FGLFNSAKLKVGVSEKTSEGCAPIVR-640
SpaC	11	647-QLKKVDAENKETELQATFE-665
SpaC	12	735-PLSKSADQGKDPNLVIL-751
SpaC	13	756-VRVGTLPKTGGHGVAIYLV-774
SodC	1	26-SSSTTTKDSADKAMTS-41
NanH	1	1-MTDSHRRGTRKALVTLTA-18
NanH	2	65-GEGKLPDPVTSEFF-78
NanH	3	520-IEDAKAATAKAEEATAN-536
NanH	4	559-AEAKSAAQDAI-569
NanH	5	595-KAENEAKALAE-605
NanH	6	617-SQDQAKALAEA-627
NanH	7	645-EKEKSGKAGGTDNTENKGFWQE-666
PknG	1	1- MNDPLSRGTEAIPFDPFADDEEDDLSGLLND-31
PknG	1.1	38-DTDTDARSREKSISTFRSRRGTNRDDRTVANG-69
PknG	1.2	79-STAEEMLKDDAYIEQKGLEKPLLHPGD-105
PknG	2	381-SPQRSTFGTKHMVFRTDQLIDGIERNVRITSEEVNA-416
PknG	3	438-YAEPSQTLQTLRDAMAQEEFANSKEIPL-465
PknG	4	479-EARSWLDTLDATLSDDWRHQWYSGVTS-505
PknG	5	576-LTKDPETLRFKALYL-590
PknG	6	627-QVPQNSTHRRMAELTAI-643
PknG	7	651-LSESRIRRAARRLESIPTNEPRFLQIKIA-679
PknG	8	718-DSLRLLARSAPNVHHRYTLV-737

^a^ Epitopes in signal peptide and conserved domains were discarded

### T-cell epitope prediction

A desirable vaccine preparation should present MHC I and II epitopes for the development of a protective and long lasting immune response to *C. pseudotuberculosis*. MHC I epitopes are presented to CD8+ T cells by cells infected with *C. pseudotuberculosis*, leading to the apoptosis of the host cell and interruption of the bacterial multiplication, and it was already described the injection of anti-CD4 or anti-CD8 monoclonal antibody resulted in significantly increased mortality and a marked suppression of IFN-gamma production in mice [53]. MHC II epitopes are involved in the activation of CD4+ T cells, which will drive the host immune response to a Th1 protective response, as well as to a production of IFN-gamma, that will help macrophages in the fusion of phagosomes and lysosomes, resulting in the destruction of bacteria that underwent phagocytic process [54]. Ultimately, specific high affinity binding should be the main concern since the efficiency of an epitope vaccine greatly relies on the precise interaction between epitope and HLA molecule [55]. Table 5 shows nine mer peptides from target proteins with high affinity (Consensus percentile rank <1 %) for mouse MHC I alleles. Most of them were from SpaC and PknG. SodC peptides were discarded since they were located in conserved regions. The few strong binding peptides to MHC II were limited to mouse H-2-IAb allele and most of them were from NanH (Table 6). Only two MHC II strong binding peptides were predicted from SodC but both were discarded because they were located in conserved regions of the protein. Additional file 4 shows the MHC class II epitopes predicted from target proteins.

**Table 5:** MHC class I epitopes predicted from target proteins

Target Protein	Mouse HLA Allele	Epitope number	Start	End	Peptide (9 mer)	Consensus rank (%)
SpaC	H-2-Db	1	615	623	FGLFNSAKL	0.3
SpaC	H-2-Kk	2	34	42	EEFENTEPI	0.3
SpaC	H-2-Kb	3	90	98	QSFNRNTGL	0.35
SpaC	H-2-Kd	4	124	132	IYAISQGRL	0.4
SpaC	H-2-Kd	5	116	124	AYNPKEGYI	0.5
SpaC	H-2-Kd	6	199	207	RYLVSNSSQ	0.5
SpaC	H-2-Kd	7	771	779	IYLVMGVLL	0.5
SpaC	H-2-Db	8	450	458	KCLQNTASI	0.6
SpaC	H-2-Db	9	208	216	SGTHNLYTL	0.7
SpaC	H-2-Dd	10	48	56	VGPSVDPTV	0.7
SpaC	H-2-Kd	11	458	466	IYANEKDLI	0.8
SpaC	H-2-Kb	12	785	793	SWSLYRNQL	0.85
SpaC	H-2-Kb	13	774	782	VMGVLLVLV	0.95
NanH	H-2-Kk	1	44	52	SEFFDSKVI	0.3
NanH	H-2-Dd	2	39	47	PDPVTSEFF	0.4
NanH	H-2-Dd	3	55	63	VDPAGQRCF	0.4
NanH	H-2-Kk	4	634	642	QELLRIFPG	0.5
NanH	H-2-Dd	5	655	663	GGMQKLLAF	0.6
NanH	H-2-Kb	6	645	653	PIFSFLASI	0.8
PknG	H-2-Kd	1	437	445	SYAEPSQTL	0.2
PknG	H-2-Kk	2	455	463	EEFANSKEI	0.2
PknG	H-2-Db	3	678	686	IAIMNAALT	0.5
PknG	H-2-Ld	4	525	533	LPGEAAPKL	0.5
PknG	H-2-Kb	5	586	594	KALYLYALV	0.55
PknG	H-2-Dd	6	665	673	SIPTNEPRF	0.6
PknG	H-2-Kb	7	685	693	LTWLRQSRL	0.6
PknG	H-2-Db	8	504	512	TSLFLDDYV	0.7
PknG	H-2-Kd	9	379	387	LYSPQRSTF	0.8
PknG	H-2-Kb	10	632	640	STHRRMAEL	0.85
PknG	H-2-Db	11	457	465	FANSKEIPL	0.9
PknG	H-2-Kk	12	21	29	EEDDLSGLL	0.9
PknG	H-2-Kk	13	353	361	LETQLFGIL	0.9

Epitopes in signal peptide and conserved domains were discarded

**Table 6:** Total numbers of MHC class II epitope prediction from target proteins

Target protein	Mouse MHC HLA allele	Number of strong binders^a^	Number of weak binders^a^	Number of peptides^b^
PknG	H-2-IAb	9	35	735
SpaC	H-2-IAb	4	48	782
SodC	H-2-IAb	0	12	192
NanH	H-2-IAb	22	64	680
PknG	H-2-IAd	0	29	735
SpaC	H-2-IAd	0	13	782
SodC	H-2-IAd	2	6	192
NanH	H-2-IAd	0	32	680

See epitope sequences in Additional file 4

^a^ Strong binder threshold 50.00. Weak binder threshold 500.00

^b^ Peptide length 15 mer

### Epitope clustering

All B and T-cell epitopes (MHC I and II) predicted from the target proteins were grouped in clusters of sequence similarity in order to evaluate the redundancy degree among them. A total of 57 clusters were formed from a set of 136 epitopes predicted (Additional file 5). Most of them (34) were single-sequence clusters. Clusters 4 and 5 (PknG) and cluster 12 (SpaC) grouped epitopes for both B and T-cell (MHC I and II). These groups of epitopes can thus potentially stimulate a complete immune response against *C. pseudotuberculosis*. The main goal of vaccination is to induce humoral and cellular immunity by selectively stimulating antigen specific CTLs or B cells together with T_H_ cells [56]. Several clusters containing B-cell and either MHC I or II epitopes were also formed. Among them are clusters 9 and 19 formed by epitopes from NanH (Additional file 5). Cluster 14 grouped all SodC weak binding epitopes to H-2-IAb allele.

### Protein expression

**Fig. 5:**
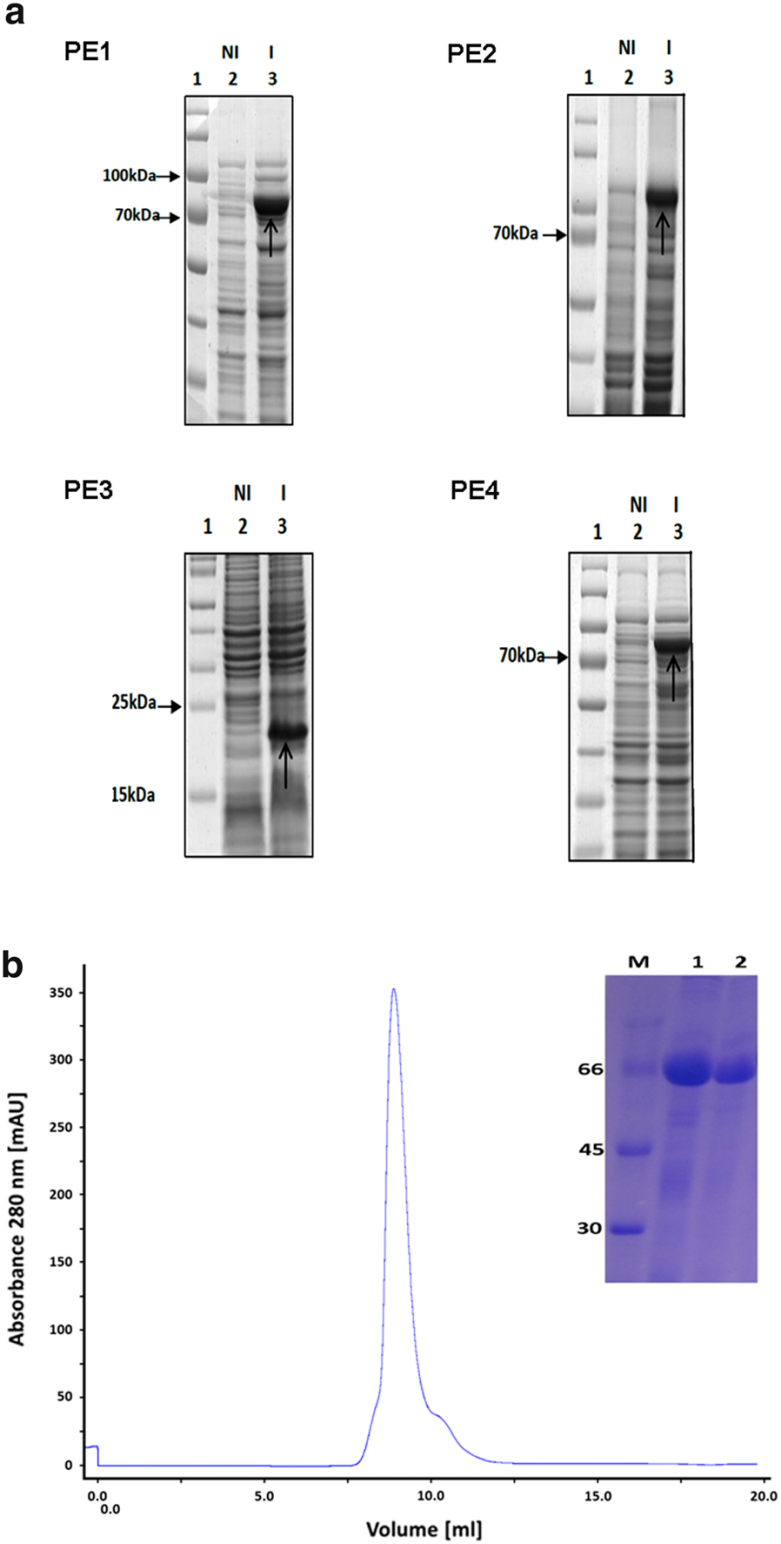
Heterologous expression of the *C. pseudotuberculosis* FRC41 putative virulence factors in *E. coli* and rPknG purification. **a** Coomassie blue-stained SDS-PAGE analyses of the protein expression experiments: PE1, rPknG expression (83 kDa, 10 % gel) in *E. coli* strain BL21 Star (DE3); PE2, rSpaC expression (86 kDa, 10 % gel) in *E. coli* strain C43 (DE3); PE3, rSodC expression (18 kDa, 15 % gel) in *E. coli* strain BL21 Star (DE3); PE4, rNanH expression (71.5 kDa, 10 % gel) in *E. coli* strain C43 (DE3). 1, pre-stained protein ladder; 2 (NI), non-induced time 0; 3 (I), induced with 1 mM IPTG for 5 h at 37 °C.* Arrows* indicate the recombinant protein position in the gels. **b** Chromatogram of the rPknG purification by gel filtration. SDS–PAGE shows an analysis of the purification steps.* M* molecular-weight markers (kDa); 1, rPknG after affinity chromatography by Ni Sepharose; 2, rPknG purified by gel filtration

Large amounts of SpaC, SodC, NanH, and PknG are necessary for future studies on the role of these proteins in *C. pseudotuberculosis* pathogenicity and virulence. *Escherichia coli* remains as one of the most attractive hosts among many systems available for heterologous protein production [57]. Thus, *pknG*, *spaC*, *sodC*, and *nanH* codon-optimized ORFs were cloned into the same expression vector system and individually transformed into BL21(DE3) *E. coli* strains. SDS-PAGE analyses show the successful expression of the target proteins (Fig. 5a). Purification of PknG using affinity and gel chromatography is shown in Fig. 5b.

From the current study we have suggested that several B and T-cell epitopes predicted from SpaC, SodC, NanH and PknG can be used for the development of a multi peptide vaccine to induce a complete immune response against *C. pseudotuberculosis*. The next step will be to evaluate experimentally these epitopes in vitro and in vivo to assess their real protective potential.

## Conclusions

The in silico analyses performed show that SpaC, PknG and NanH present good potential as targets for vaccine development. Several epitopes from these proteins can potentially induce both humoral and cellular immune responses against *C. pseudotuberculosis*. The four target proteins were successfully expressed in *E. coli*. The production of these proteins in large amounts represents an important step for future studies on 3-D structure, pathogenicity, virulence, and vaccine development.

## Additional file

10.1186/s12934-016-0479-6 Homologous proteins of *C. pseudotuberculosis* FRC41 putative virulence factor PknG in CMNR microorganism and mammalians.
10.1186/s12934-016-0479-6 Homologous proteins of *C. pseudotuberculosis* FRC41 putative virulence factors SpaC and NanH in CMNR microorganism and mammalians.
10.1186/s12934-016-0479-6 Homologous proteins of *C. pseudotuberculosis* FRC41 putative virulence factor SodC in CMNR microorganism and mammalians.
10.1186/s12934-016-0479-6 MHC class II epitopes§ predicted from target proteins.
10.1186/s12934-016-0479-6 Clusters of B and T-cell epitopes predicted from target proteins.

## Abbreviations

CLA: caseous lymphadenitis
CMNR: microorganisms from *Corynebacterium*, *Mycobacterium*, *Nocardi,* and *Rhodococcus* genera
PLD: phospholipase D
SOD: superoxide dismutase
ORF: open reading frame
SDS-PAGE: sodium dodecyl sulfate polyacrylamide gel electrophoresis
