# Supplementary material for: Putative virulence factors of Corynebacterium pseudotuberculosis FRC41: vaccine potential and protein expression
Source: Microb Cell Fact. 2016 May 16;15:83. doi: 10.1186/s12934-016-0479-6 (PMC4869379; doi:10.1186/s12934-016-0479-6)
Supplement: Supplementary file 1 — 10.1186/s12934-016-0479-6 Homologous proteins of C. pseudotuberculosis FRC41 putative virulence factor PknG in CMNR microorganism and mammalians. [file 12934_2016_479_MOESM1_ESM.pdf]

Homologous proteins in CMNR[1] microorganisms				Homologous proteins in Mammalians[2]			
**Organism**	**Protein**	**Identity (%)**	**E-value**	**Organism**	**Protein**	**Identity (%)**	**E-value**
C. pseudotuberculosis 1002	PknG	100	0.0	*Ovis aries*	Uncharacterized Protein PRKX[4]	30	4.0×10^{-12}
C. pseudotuberculosis 258	PknG	99	0.0	*Ovis aries*	Uncharacterized Protein CAMK1[5]	29	4.0×10^{-12}
C. pseudotuberculosis C231	PknG	100	0.0	*Bos Taurus*	Uncharacterized Protein PRKX[4]	30	3.0×10^{-12}
C. diphteriae HC02	PknG	74	0.0	*Bos Taurus*	CAMK1[5]	29	1.0×10^{-12}
C. glutamicum ATCC 14067	S/T PK[3]	61	0.0	*Equs caballus*	Uncharacterized Protein CAMK1[5]	31	9.0×10^{-13}
M. tuberculosis SUMu007	PknG	46	1.0×10^{-169}	*Mus musculus*	CAMKI[5]	29	3×10^{-13}
N. farcinica IFM10152	Putative S/T PK[3]	41	4.0×10^{-173}	*Mus musculus*	Smok2b[6]	31	1×10^{-13}
R. pyridinivorans SB3094	S/T PK[3]	46	1.0×10^{-179}	*Homo sapiens*	CAMK1[5]	29	2×10^{-12}

Additional file 1: NCBI BLASTP searches were performed in UniprotKB database. [1]*Corynebacterium, Mycobacterium, Nocardia, Rhodococcus*; [2]*Ovis, Bos, Equus and Mus* genera, *Homo sapiens*; [3]S/T PK, serine/threonine protein kinase; [4]PRKX, protein kinase X-linked; [5]CAMK1, Calcium/calmodulin-dependent protein kinase 1; [6]Smok2b, sperm motility kinase 2B.
