# Supplementary material for: Putative virulence factors of Corynebacterium pseudotuberculosis FRC41: vaccine potential and protein expression
Source: Microb Cell Fact. 2016 May 16;15:83. doi: 10.1186/s12934-016-0479-6 (PMC4869379; doi:10.1186/s12934-016-0479-6)
Supplement: Supplementary file 2 — 10.1186/s12934-016-0479-6 Homologous proteins of C. pseudotuberculosis FRC41 putative virulence factors SpaC and NanH in CMNR microorganism and mammalians. [file 12934_2016_479_MOESM2_ESM.pdf]

SpaC homologous proteins in CMNR[1] microorganisms				SpaC homologous proteins in mammalians[2]			
**Organism**	**Protein**	**Identity (%)**	**E-value**	**Organism**	**Protein**	**Identity (%)**	**E-value**
C. pseudotuberculosis 1002	Uncharacterized protein	99	0.0	No significant similarity found.			
C. pseudotuberculosis C231	Uncharacterized protein	100	0.0				
C. diphteriae HC02	Putative fimbrial subunit	27	$5x10^{-15}$				
**NanH homologous proteins in CMNR[1] microorganisms**				**NanH homologous proteins in mammalians[2]**			
**Organism**	**Protein**	**Identity (%)**	**E-value**	**Organism**	**Protein**	**Identity (%)**	**E-value**
C. pseudotuberculosis 1002	NanH	100	0.0	*Bos taurus*	Plectin	30%	$1x10^{-8}$
C. pseudotuberculosis C231	NanH	100	0.0	*Mus musculus*	Plectin	30%	$2x10^{-8}$
C. diphteriae HC02	NanH	50	0.0	*Homo sapiens*	Plectin	30%	$2x10^{-9}$
C. glutamicum ATCC 14067	Uncharacterized protein	36	$1.0x10^{-56}$	*Homo sapiens*	NEU[3]	27%	$2x10^{-7}$

Additional file 2: NCBI BLASTP searches were performed in UniprotKB database. [1]*Corynebacterium, Mycobacterium, Nocardia, Rhodococcus*; [2]*Ovis*, *Bos*, *Equus and Mus* genera, *Homo sapiens*; [3]Sialidase 1 (lysosomal sialidase).
