# Supplementary material for: Putative virulence factors of Corynebacterium pseudotuberculosis FRC41: vaccine potential and protein expression
Source: Microb Cell Fact. 2016 May 16;15:83. doi: 10.1186/s12934-016-0479-6 (PMC4869379; doi:10.1186/s12934-016-0479-6)
Supplement: Supplementary file 3 — 10.1186/s12934-016-0479-6 Homologous proteins of C. pseudotuberculosis FRC41 putative virulence factor SodC in CMNR microorganism and mammalians. [file 12934_2016_479_MOESM3_ESM.pdf]

Homologous proteins in CMNR[1] microorganisms				Homologous proteins in mammalians[2]			
**Organism**	**Protein**	**Identity (%)**	**E-value**	**Organism**	**Protein**	**Identity (%)**	**E-value**
C. pseudotuberculosis 1002	SodC	100	$3x10^{-122}$	*Ovis aries*	Sod	36%	$3x10^{-15}$
C. pseudotuberculosis 258	SodC	100	$3x 10^{-122}$	*Bos taurus*	Sod1	34%	$2x10^{-15}$
C. pseudotuberculosis C231	SodC	100	$3x10^{-122}$	*Equs caballus*	Sod1	32%	$8x10^{-15}$
C. diphteriae HC02	SodC	68	$2x10^{-75}$	*Mus musculus*	Sod1	34%	$2x10^{-16}$
M. tuberculosis SUMu007	SodC	43	$9x10^{-28}$	*Homo sapiens*	Sod1	34%	$3x10^{-16}$
N. farcinica IFM 10152	SodC	44	$4x10^{-35}$				
Rhodococcus sp. RHA1	SodC	49	$2x10^{-42}$				

Additional file 3: NCBI BLASTP searches were performed in UniprotKB database. [1]*Corynebacterium, Mycobacterium, Nocardia, Rhodococcus*; [2]*Ovis, Bos, Equus and Mus* genera, *Homo sapiens*.
