# Supplementary material for: Putative virulence factors of Corynebacterium pseudotuberculosis FRC41: vaccine potential and protein expression
Source: Microb Cell Fact. 2016 May 16;15:83. doi: 10.1186/s12934-016-0479-6 (PMC4869379; doi:10.1186/s12934-016-0479-6)
Supplement: Supplementary file 4 — 10.1186/s12934-016-0479-6 MHC class II epitopes§ predicted from target proteins. [file 12934_2016_479_MOESM4_ESM.pdf]

Target Protein	Mouse MHC II Allele	Epitope number	Peptide*	Core	1-log50k (aff)	Affinity (nM)	Rank (%)	Level of binding**
PknG	H-2-IAb	1	DGVYLPGIPSPDEEK	YLPGIPSPD	0.6959	26.8	0.20	SB
SpaC	H-2-IAb	1	LDEFKPVGARTPWVY	FKPVGARTP	0.6740	34.0	0.40	SB
SpaC	H-2-IAb	2	GLDEFKPVGARTPWV	FKPVGARTP	0.6725	34.6	0.40	SB
SpaC	H-2-IAb	3	TGLDEFKPVGARTPW	FKPVGARTP	0.6615	39.0	0.40	SB
SpaC	H-2-IAb	4	NTGLDEFKPVGARTP	FKPVGARTP	0.6460	46.1	0.50	SB
SodC	H-2-IAb	01	ADKAMTSAASTASNS	AMTSAASTA	0.5721	102.5	1.50	WB
SodC	H-2-IAb	1	DKAMTSAASTASNSH	MTSAASTAS	0.5604	116.3	2.00	WB
SodC	H-2-IAb	1.1	SADKAMTSAASTASN	AMTSAASTA	0.5585	118.7	2.00	WB
SodC	H-2-IAb	1.2	DSADKAMTSAASTAS	AMTSAASTA	0.5417	142.3	4.00	WB
SodC	H-2-IAb	2	KAMTSAASTASNSHA	AMTSAASTA	0.5399	145.2	4.00	WB
SodC	H-2-IAb	3	AMTSAASTASNSHAF	AMTSAASTA	0.4993	225.2	4.00	WB
SodC	H-2-IAb	3.1	KDSADKAMTSAASTA	AMTSAASTA	0.4719	303.2	8.00	WB

SodC	H-2-IAb	4	MTSAASTASNSHAFA	SAASTASNS	0.4372	440.9	8.00	WB
NanH	H-2-IAb	1	NGLFDAAPPAPVARG	FDAAPPAPV	0.7173	21.3	0.15	SB
NanH	H-2-IAb	2	NNGLFDAAPPAPVAR	FDAAPPAPV	0.7109	22.8	0.15	SB
NanH	H-2-IAb	3	GLFDAAPPAPVARGA	FDAAPPAPV	0.7108	22.8	0.15	SB
NanH	H-2-IAb	4	DNNGLFDAAPPAPVA	FDAAPPAPV	0.6956	26.9	0.20	SB
NanH	H-2-IAb	5	LFDAAPPAPVARGAV	FDAAPPAPV	0.6905	28.5	0.30	SB
NanH	H-2-IAb	6	IDNNGLFDAAPPAPV	FDAAPPAPV	0.6665	36.9	0.40	SB
NanH	H-2-IAb	7	FDAAPPAPVARGAVG	FDAAPPAPV	0.6587	40.1	0.40	SB
PknG	H-2-IAd	1	TWLRQSRLEAAAADN	LRQSRLEAA	0.5270	167.0	0.80	WB
PknG	H-2-IAd	2	WLRQSRLEAAAADND	LRQSRLEAA	0.5231	174.2	0.80	WB
PknG	H-2-IAd	3	STHRRMAELTAILHL	RRMAELTAI	0.5186	182.8	0.90	WB
PknG	H-2-IAd	4	THRRMAELTAILHLI	RRMAELTAI	0.5129	194.4	1.00	WB
PknG	H-2-IAd	5	LTWLRQSRLEAAAAD	LRQSRLEAA	0.5074	206.3	1.00	WB
PknG	H-2-IAd	6	PRFLQIKIAIMNAAL	PRFLQIKIA	0.5041	213.9	1.10	WB
PknG	H-2-IAd	7	EPRFLQIKIAIMNAA	PRFLQIKIA	0.4959	233.8	1.25	WB

PknG	H-2-IAd	8	HRRMAELTAILHLIS	RRMAELTAI	0.4932	240.7	1.25	WB
PknG	H-2-IAd	9	LRQSRLEAAAADNDL	LRQSRLEAA	0.4882	254.0	1.50	WB
PknG	H-2-IAd	10	NEPRFLQIKIAIMNA	PRFLQIKIA	0.4690	312.8	2.00	WB
PknG	H-2-IAd	11	NSTHRRMAELTAILH	RRMAELTAI	0.4672	319.0	2.00	WB
PknG	H-2-IAd	12	ARSREKSISTFRSRR	SREKSISTF	0.4631	333.2	2.00	WB
PknG	H-2-IAd	13	RSREKSISTFRSRRG	SREKSISTF	0.4540	367.9	4.00	WB
PknG	H-2-IAd	14	RRMAELTAILHLISG	RRMAELTAI	0.4516	377.5	4.00	WB
PknG	H-2-IAd	15	ALTWLRQSRLEAAAA	LRQSRLEAA	0.4504	382.6	4.00	WB
PknG	H-2-IAd	16	EEVNAALPVPLLDRT	NAALPVPLL	0.4470	396.8	4.00	WB
PknG	H-2-IAd	17	DARSREKSISTFRSR	SREKSISTF	0.4365	444.3	4.00	WB
PknG	H-2-IAd	18	ANGMVTLPFIPVSTA	NGMVTLPFI	0.4365	444.7	4.00	WB
PknG	H-2-IAd	19	SREKSISTFRSRRGT	SREKSISTF	0.4307	473.3	4.00	WB
PknG	H-2-IAd	20	FANSKEIPLGVVRTL	SKEIPLGVV	0.4296	479.1	4.00	WB
PknG	H-2-IAd	21	EVNAALPVPLLDRTD	NAALPVPLL	0.4274	490.6	4.00	WB
SpaC	H-2-IAd	1	GKKNQEVQAADISVN	KKNQEVQAA	0.5410	143.4	0.60	WB

SpaC	H-2-IAd	2	YGKKNQEVQAADISV	KKNQEVQAA	0.5306	160.6	0.70	WB
SpaC	H-2-IAd	3	DYGKKNQEVQAADIS	KKNQEVQAA	0.5194	181.3	0.90	WB
SpaC	H-2-IAd	4	KKNQEVQAADISVNG	KKNQEVQAA	0.5140	192.1	0.90	WB
SpaC	H-2-IAd	5	GKADVTIDAASFPLS	KADVTIDAA	0.4886	253.0	1.50	WB
SpaC	H-2-IAd	6	LFNSAKLKVGVSEKT	SAKLKVGVS	0.4774	285.4	2.00	WB
SpaC	H-2-IAd	7	ADGKADVTIDAASFP	KADVTIDAA	0.4742	295.6	2.00	WB
SpaC	H-2-IAd	8	FNSAKLKVGVSEKTS	SAKLKVGVS	0.4727	300.4	2.00	WB
SpaC	H-2-IAd	9	DGKADVTIDAASFPL	KADVTIDAA	0.4710	306.0	2.00	WB
SpaC	H-2-IAd	10	NSAKLKVGVSEKTSE	SAKLKVGVS	0.4699	309.7	2.00	WB
SpaC	H-2-IAd	11	SAKLKVGVSEKTSEG	SAKLKVGVS	0.4591	348.2	4.00	WB
SpaC	H-2-IAd	12	TADGKADVTIDAASF	KADVTIDAA	0.4439	410.5	4.00	WB
SpaC	H-2-IAd	13	SDYGKKNQEVQAADI	KKNQEVQAA	0.4429	414.8	4.00	WB
NanH	H-2-IAd	1	EAKSAAQDAIAAAEE	SAAQDAIAA	0.5294	162.6	0.70	WB
NanH	H-2-IAd	2	AEAKSAAQDAIAAAE	EAKSAAQDA	0.5199	180.2	0.90	WB
NanH	H-2-IAd	3	LAEQLAKVEAELANS	LAKVEAELA	0.5049	212.2	1.10	WB

NanH	H-2-IAd	4	AEQLAKVEAELANSQ	LAKVEAELA	0.5028	216.8	1.10	WB
NanH	H-2-IAd	5	ALAEQLAKVEAELAN	LAKVEAELA	0.4935	239.9	1.25	WB
NanH	H-2-IAd	6	AEAKEAAEIARKAAE	AAEIARKAA	0.4933	240.5	1.25	WB
NanH	H-2-IAd	7	LAEAKSAAQDAIAAA	EAKSAAQDA	0.4901	249.0	1.50	WB
NanH	H-2-IAd	8	AKSAAQDAIAAAEEA	AAQDAIAAA	0.4774	285.7	2.00	WB
NanH	H-2-IAd	9	LAEAKEAAEIARKAA	EAKEAAEIA	0.4743	295.3	2.00	WB
NanH	H-2-IAd	10	AKEAAEIARKAAEEA	AAEIARKAA	0.4631	333.4	2.00	WB
NanH	H-2-IAd	11	EQLAKVEAELANSQD	LAKVEAELA	0.4620	337.2	4.00	WB
NanH	H-2-IAd	12	EAKEAAEIARKAAEE	AAEIARKAA	0.4599	344.9	4.00	WB
NanH	H-2-IAd	13	QLAKVEAELANSQDQ	LAKVEAELA	0.4536	369.5	4.00	WB
NanH	H-2-IAd	15	KALAEQLAKVEAELA	LAKVEAELA	0.4493	387.0	4.00	WB
NanH	H-2-IAd	16	AAEIARKAAEEALKL	AAEIARKAA	0.4404	426.0	4.00	WB
NanH	H-2-IAd	17	KALAEAKEAAEIARK	KALAEAKEA	0.4397	429.4	4.00	WB
NanH	H-2-IAd	18	KSAAQDAIAAAEEAN	AAQDAIAAA	0.4372	441.0	4.00	WB
NanH	H-2-IAd	20	GATIEAEEAAMKAEN	GATIEAEEA	0.4341	456.1	4.00	WB

NanH	H-2-IAd	21	EAAEIARKAAEEALK	AAEIARKAA	0.4323	465.0	4.00	WB

Additional file 4: [§]Epitopes in signal peptide and conserved domains were discarded. [*]Peptide length 15 mer. [**]Strong binder (SB) threshold 50.00. Weak binder (WB) threshold 500.00.
