# Supplementary material for: Putative virulence factors of Corynebacterium pseudotuberculosis FRC41: vaccine potential and protein expression
Source: Microb Cell Fact. 2016 May 16;15:83. doi: 10.1186/s12934-016-0479-6 (PMC4869379; doi:10.1186/s12934-016-0479-6)
Supplement: Supplementary file 5 — 10.1186/s12934-016-0479-6 Clusters of B and T-cell epitopes predicted from target proteins. [file 12934_2016_479_MOESM5_ESM.pdf]

Additional file 5:

Cluster number	Number of epitopes in the cluster	Number of epitopes	Target-protein/epitope/number	Epitope sequence
		1	SpaC/MHC-I_H-2-Kb/13	VMGVLLVLV
1	3	2	SpaC/MHC-I_H-2-Kd/7	IYLVMGVLL
		3	SpaC/B-cell/13	VRVGTLPKTGGHGVAIYLV
2	2	1	PknG/MHC-II_H-2-IAd/21	EEVNAALPVPLLDRT
		2	PknG/MHC-II_H-2-IAd/29	EVNAALPVPLLDRTD
		1	SpaC/MHC-I_H-2-Kd/5	AYNPKEGYI
3	3	2	SpaC/MHC-I_H-2-Kd/4	IYAISQGRL
		3	SpaC/B-cell/3	AYNPKEGYIYAISQGRLKTLQSSK LRIYDEDPNYPAGHLL
		1	PknG/MHC-II_H-2-IAd/28	FANSKEIPLGVVRTL
4	4	2	PknG/MHC-I_H-2-Db/11	FANSKEIPL
		3	PknG/B-cell/3	YAEPSQTLQTLRDAMAQEEFANS KEIPL

		4	PknG/MHC-I_H-2-Kk/2	EEFANSKEI
		1	PknG/MHC-II_H-2-IAd/19	RRMAELTAILHLISG
		2	PknG/MHC-II_H-2-IAd/8	HRRMAELTAILHLIS
		3	PknG/MHC-II_H-2-IAd/4	THRRMAELTAILHLI
5	7	4	PknG/MHC-II_H-2-IAd/15	NSTHRRMAELTAILH
		5	PknG/MHC-II_H-2-IAd/3	STHRRMAELTAILHL
		6	PknG/B-cell/6	QVPQNSTHRRMAELTAI
		7	PknG/MHC-I_H-2-Kb/10	STHRRMAEL
		1	SpaC/MHC-II_H-2-IAd/4	KKNQEVQAADISVNG
		2	SpaC/MHC-II_H-2-IAd/1	GKKNQEVQAADISVN
		3	SpaC/MHC-II_H-2-IAd/13	SDYGKKNQEVQAADI
6	6	4	SpaC/MHC-II_H-2-IAd/3	DYGKKNQEVQAADIS
		5	SpaC/B-cell/9	KLERLKVISDYGKKNQEVQAADI SV
		6	SpaC/MHC-II_H-2-IAd/2	YGKKNQEVQAADISV
7	5	1	PknG/MHC-II_H-2-IAd/24	DARSREKSISTFRSR

		2	PknG/MHC-II_H-2-IAd/16	ARSREKSISTFRSRR
		3	PknG/MHC-II_H-2-IAd/27	SREKSISTFRSRRGT
		4	PknG/B-cell/1.1	DTDTDARSREKSISTFRSRRGTNR DDRTVANG
		5	PknG/MHC-II_H-2-IAd/18	RSREKSISTFRSRRG
8	4	1	PknG/MHC-II_H-2-IAd/14	NEPRFLQIKIAIMNA
		2	PknG/MHC-II_H-2-IAd/6	PRFLQIKIAIMNAAL
		3	PknG/MHC-II_H-2-IAd/7	EPRFLQIKIAIMNAA
		4	PknG/MHC-I_H-2-Db/3	IAIMNAALT
9	6	1	NanH/MHC-II_H-2-IAd/23	KSAAQDAIAAAEEAN
		2	NanH/MHC-II_H-2-IAd/9	AKSAAQDAIAAAEEA
		3	NanH/MHC-II_H-2-IAd/1	EAKSAAQDAIAAAEE
		4	NanH/MHC-II_H-2-IAd/7	LAEAKSAAQDAIAAA
		5	NanH/B-cell/4	AEAKSAAQDAI
		6	NanH/MHC-II_H-2-IAd/2	AEAKSAAQDAIAAAE
10	6	1	NanH/MHC-II_H-2-IAd/19	KALAEQLAKVEAELA

		2	NanH/MHC-II_H-2-IAd/17	QLAKVEAELANSQDQ
		3	NanH/MHC-II_H-2-IAd/15	EQLAKVEAELANSQD
		4	NanH/MHC-II_H-2-IAd/5	ALAEQLAKVEAELAN
		5	NanH/MHC-II_H-2-IAd/3	LAEQLAKVEAELANS
		6	NanH/MHC-II_H-2-IAd/4	AEQLAKVEAELANSQ
		1	NanH/MHC-II_H-2-IAd/21	AAEIARKAAEEALKL
		2	NanH/MHC-II_H-2-IAd/27	EAAEIARKAAEEALK
		3	NanH/MHC-II_H-2-IAd/22	KALAEAKEAAEIARK
11	7	4	NanH/MHC-II_H-2-IAd/6	AEAKEAAEIARKAAE
		5	NanH/MHC-II_H-2-IAd/10	LAEAKEAAEIARKAA
		6	NanH/MHC-II_H-2-IAd/14	AKEAAEIARKAAEEA
		7	NanH/MHC-II_H-2-IAd/16	EAKEAAEIARKAAEE
		1	SpaC/MHC-II_H-2-IAd/11	SAKLKVGVSEKTSEG
12	6	2	SpaC/MHC-II_H-2-IAd/10	NSAKLKVGVSEKTSE
		3	SpaC/MHC-II_H-2-IAd/8	FNSAKLKVGVSEKTS

		4	SpaC/MHC-II_H-2-IAd/6	LFNSAKLKVGVSEKT
		5	SpaC/B-cell/10	FGLFNSAKLKVGVSEKTSEGCAPIVR
		6	SpaC/MHC-I_H-2-Db/1	FGLFNSAKL
13	5	1	SpaC/MHC-II_H-2-IAb/4	NTGLDEFKPVGARTP
		2	SpaC/MHC-II_H-2-IAb/3	TGLDEFKPVGARTPW
		3	SpaC/MHC-II_H-2-IAb/1	LDEFKPVGARTPWVY
		4	SpaC/MHC-II_H-2-IAb/2	GLDEFKPVGARTPWV
		5	SpaC/MHC-I_H-2-Kb/3	QSFNRNTGL
14	8	1	SodC/ MHC-II_H-2-IAb/01	ADKAMTSAASTASNS
		2	SodC/ MHC-II_H-2-IAb/1	DKAMTSAASTASNSH
		3	SodC/ MHC-II_H-2-IAb/1.1	SADKAMTSAASTASN
		4	SodC/ MHC-II_H-2-IAb/1.2	DSADKAMTSAASTAS
		5	SodC/ MHC-II_H-2-IAb/2	KAMTSAASTASNSHA
		6	SodC/ MHC-II_H-2-IAb/3	AMTSAASTASNSHAF
		7	SodC/ MHC-II_H-2-IAb/3.1	KDSADKAMTSAASTA

		8	SodC/ MHC-II_H-2-IAb/4	MTSAASTASNSHAFA
15	4	1	SpaC/MHC-II_H-2-IAd/12	TADGKADVTIDAASF
		2	SpaC/MHC-II_H-2-IAd/7	ADGKADVTIDAASFP
		3	SpaC/MHC-II_H-2-IAd/5	GKADVTIDAASFPLS
		4	SpaC/MHC-II_H-2-IAd/9	DGKADVTIDAASFPL
16	6	1	PknG/MHC-II_H-2-IAd/20	ALTWLRQSRLEAAAA
		2	PknG/MHC-I_H-2-Kb/7	LTWLRQSRL
		3	PknG/MHC-II_H-2-IAd/5	LTWLRQSRLEAAAAD
		4	PknG/MHC-II_H-2-IAd/9	LRQSRLEAAAADNDL
		5	PknG/MHC-II_H-2-IAd/1	TWLRQSRLEAAAADN
		6	PknG/MHC-II_H-2-IAd/2	WLRQSRLEAAAADND
17	7	1	NanH/MHC-II_H-2-IAb/6	NNGLFDAAPPAPVAR
		2	NanH/MHC-II_H-2-IAb/4	NGLFDAAPPAPVARG
		3	NanH/MHC-II_H-2-IAb/7	GLFDAAPPAPVARGA
		4	NanH/MHC-II_H-2-IAb/9	DNNGLFDAAPPAPVA

		5	NanH/MHC-II_H-2-IAb/17	IDNNGLFDAAPPAPV
		6	NanH/MHC-II_H-2-IAb/19	FDAAPPAPVARGAVG
		7	NanH/MHC-II_H-2-IAb/10	LFDAAPPAPVARGAV
18	2	1	PknG/B-cell/7	LSESRIRRAARRLESIPTNEPRFLQI KIA
		2	PknG/MHC-I_H-2-Dd/6	SIPTNEPRF
19	2	1	NanH/B-cell/2	GEGKLPDPVTSEFF
		2	NanH/MHC-I_H-2-Dd/2	PDPVTSEFF
20	3	1	SpaC/B-cell/7	KATYKVTANQSISNNEKCLQNTA SIYAN
		2	SpaC/MHC-I_H-2-Db/8	KCLQNTASI
		3	SpaC/MHC-I_H-2-Kd/11	IYANEKDLI
21	2	1	PknG/B-cell/2	SPQRSTFGTKHMVFRTDQLIDGIE RNVRITSEEVNA
		2	PknG/MHC-I_H-2-Kd/9	LYSPQRSTF
22	2	1	PknG/MHC-I_H-2-Kk/12	EEDDLSGLL
		2	PknG/B-cell/1	MNDPLSRGTEAIPFDPFADDEEDD

				LSGLLND
23	2	1	PknG/B-cell/5	LTKDPETLRFKALYL
		2	PknG/MHC-I_H-2-Kb/5	KALYLYALV
24	1	1	NanH/B-cell/3	IEDAKAATAKAEEATAN
25	1	1	NanH/B-cell/7	EKEKSGKAGGTDNTENKGFWQE
26	1	1	SpaC/B-cell/6	IVAKRKGPTSQNNDATSNG
27	1	1	NanH/MHC-I_H-2-Dd/5	GGMQKLLAF
28	1	1	NanH/MHC-I_H-2-Dd/3	VDPAGQRCF
29	1	1	NanH/MHC-I_H-2-Kk/4	QELLRIFPG
30	1	1	SodC/B-cell/1	SSSTTTKDSADKAMTS
31	1	1	SpaC/B-cell/4	NDYTSTGKTDSNYVWGI
32	1	1	PknG/MHC-I_H-2-Db/8	TSLFLDDYV
33	1	1	SpaC/B-cell/5	KNSSNPAVLERIDVRDGSRKEFSL DGVKDPLGQNVEKGIYGT
34	1	1	PknG/MHC-I_H-2-Kk/13	LETQLFGIL
35	1	1	SpaC/B-cell/2	SQHTNRGETFNDRNSTDLYVQ

36	1	1	NanH/MHC-II_H-2-IAd/26	GATIEAEEAAMKAEN
37	1	1	NanH/B-cell/5	KAENEAKALAE
38	1	1	NanH/B-cell/6	SQDQAKALAEA
39	1	1	SpaC/MHC-I_H-2-Kd/6	RYLVSNSSQ
40	1	1	SpaC/MHC-I_H-2-Kb/12	SWSLYRNQL
41	1	1	NanH/B-cell/1	MTDSHRRGTRKALVTLTA
42	1	1	SpaC/MHC-I_H-2-Db/9	SGTHNLYTL
43	1	1	PknG/MHC-I_H-2-Kd/1	SYAEPSQTL
44	1	1	PknG/B-cell/8	DSLRLLARSAPNVHHRYTLV
45	1	1	PknG/MHC-I_H-2-Ld/4	LPGEAAPKL
46	1	1	PknG/MHC-II_H-2-IAb/1	DGVYLPGIPSPDEEK
47	1	1	PknG/B-cell/1.2	STAEEMLKDDAYIEQKGLEKPLL HPGD
48	1	1	SpaC/B-cell/8	GNGLRKVTYKIEVKNPKGFPETK YSLTDTPQFADSV
49	1	1	PknG/MHC-II_H-2-IAd/25	ANGMVTLPFIPVSTA

50	1	1	NanH/MHC-I_H-2-Kb/6	PIFSFLASI
51	1	1	PknG/B-cell/4	EARSWLDTLDATLSDDWRHQWY SGVTS
52	1	1	SpaC/B-cell/11	QLKKVDAENKETELQATFE
53	1	1	SpaC/B-cell/12	PLSKSADQGKDPNLVIL
54	1	1	SpaC/MHC-I_H-2-Dd/10	VGPSVDPTV
55	1	1	SpaC/B-cell/1	MEVPEKTKVEIRFQTGSKISTPSTP SV
56	1	1	SpaC/MHC-I_H-2-Kk/2	EEFENTEPI
57	1	1	NanH/MHC-I_H-2-Kk/1	SEFFDSKVI
